# Rates and Risk Factors for Coccidioidomycosis among Prison Inmates, California, USA, 2011

**DOI:** 10.3201/eid2101.140836

**Published:** 2015-01

**Authors:** Charlotte Wheeler, Kimberley D. Lucas, Janet C. Mohle-Boetani

**Affiliations:** California Correctional Health Care Services, Elk Grove, California, USA

**Keywords:** African American, black, white, race, ethnicity, coccidioides, coccidioidomycosis, diabetes mellitus, Hispanic American, lung diseases, fungal epidemiology, mycoses, fungus, prisons, prisoners, inmates, valley fever, cocci

## Abstract

In California, coccidioidomycosis is a disease acquired by inhaling spores of *Coccidioides immitis*, a fungus found in certain arid regions, including the San Joaquin Valley, California, USA, where 8 state prisons are located. During 2011, we reviewed coccidioidomycosis rates at 2 of the prisons that consistently report >80% of California’s inmate cases and determined inmate risk factors for primary, severe (defined as pulmonary coccidioidomycosis requiring >10 hospital days), and disseminated coccidioidomycosis (defined by hospital discharge International Classification of Disease, Ninth Revision code). Inmates of African American ethnicity who were >40 years of age were at significantly higher risk for primary coccidioidomycosis than their white counterparts (odds ratio = 2.0, 95% CI 1.5–2.8). Diabetes was a risk factor for severe pulmonary coccidioidomycosis, and black race a risk factor for disseminated disease. These findings contributed to a court decision mandating exclusion of black inmates and inmates with diabetes from the 2 California prisons with the highest rates of coccidioidomycosis.

## Abstract 2

African American ethnicity is a risk factor for primary and disseminated disease in this population.

Coccidioidomycosis, commonly called “cocci” or “valley fever,” is an illness caused by *Coccidioides immitis* and *C. posadasii*, soil-dwelling fungi found in certain arid regions of the southwestern United States, northern Mexico, and Central and South America. Infection is acquired by inhaling airborne fungal spores and is not spread person-to-person. Sixty percent of *Coccidioides* infections are asymptomatic, and most symptomatic infections consist of self-limited, flu-like illnesses. A small proportion of cases result in prolonged illness that may require lifelong treatment and can be life-threatening, particularly the 3%–5% in which the disease disseminates outside of the lungs. Infection, except in very rare cases, confers lifelong immunity.

In 2005, the medical executive team of the California Department of Corrections and Rehabilitation (CDCR) informed the California Department of Public Health (CDPH) that physicians at 2 prisons for adult men (prison X and prison Y) reported an increase in the number of inmates with coccidioidomycosis. The prisons are located <15 miles apart from one another in a *Coccidioides*-endemic area of California’s San Joaquin Valley. In response to the call, CDPH investigated the cases at prison X and confirmed rates of disease >400× higher than those of the surrounding county. Additionally, CDPH performed a cohort study at prison X and identified an increased risk for coccidioidomycosis among African-American inmates, inmates >40 years of age, and inmates who resided on a particular yard (J. Yuan, unpub. data).

In 2006, CDPH made recommendations concerning coccidioidomycosis. In response, the California Correctional Health Care Services (CCHCS) (the medical arm for California inmates) instituted policies for educating inmates and staff about coccidioidomycosis and for excluding inmates with immunocompromising conditions or severe chronic obstructive pulmonary disease from California prisons in 3 coccidioidomycosis-endemic counties. In addition, the agency mandated the cancellation of planned construction to expand prison X. During subsequent years, prisons X and Y took measures to control ambient dust (and presumably spores) by planting native grasses and shrubs on bare grounds. In December 2011, prison X applied a soil-stabilizing emulsion to most of the grounds within the prison’s perimeter. Despite these efforts, high coccidioidomycosis attack rates continued to be reported from these institutions (CCHCS coccidioidomycosis surveillance system, unpub. data).

The purpose of this study was to review rates of coccidioidomycosis at prisons X and Y, to reevaluate the population for risk factors for development of primary disease, as well as to evaluate inmate risk factors for development of the most debilitating forms of coccidioidomycosis. We used the study results to improve the policies and practices for protecting California inmates from coccidioidomycosis and its most serious sequelae.

## Materials and Methods

### Coccidioidomycosis Incidence and Cases per Person-Years

We calculated coccidioidomycosis incidence in 2 ways: 1) as a proportion of the population at risk, and 2) as the number of cases per person-years. Because community coccidioidomycosis rates are measured by incidence proportions, we calculated inmate rates by the same measurement to enhance comparison. Because cases per person-years is the recommended measure of disease incidence in a dynamic population, and because inmates are frequently moved from one prison to another throughout a year, calculating inmate cases per person-years gave us a measure against which to check coccidioidomycosis incidence proportions. Our concern was that the coccidioidomycosis incidence proportions might overestimate coccidioidomycosis rates in this study population.

To calculate coccidioidomycosis incidence proportion in the prisons, we derived coccidioidomycosis case counts from a surveillance system implemented in California prisons in 2007. Public health nurses assigned to CDCR prisons report coccidioidomycosis cases to the CCHCS Public Health Branch. Cases must meet the National Notifiable Diseases Surveillance System case definition for coccidioidomycosis (*1*). We calculated the yearly rates in prisons by dividing the surveillance-derived case counts by the published mid-year inmate populations (*2*). We obtained city coccidioidomycosis counts from local county health departments (F. Aranki, M. MacLean, unpub. data), and county and state coccidioidomycosis counts from data published by CDPH (*3*). We calculated annual community rates by dividing coccidioidomycosis counts by mid-year population estimates obtained from local health departments (for cities) and from the California Department of Finance (for counties and the state of California) (*4*,*5*). Because community data contain prison counts, prisons X and Y coccidioidomycosis counts were subtracted from their respective community coccidioidomycosis counts (city, county, and state counts), and prisons X and Y population counts were subtracted from their respective community populations. We compared prisons X and Y coccidioidomycosis incidence proportions to the incidence proportions of their surrounding communities and to those of Kern County and the state of California. Kern County coccidioidomycosis incidence proportions are benchmarks because Kern County consistently reports the highest coccidioidomycosis incidence of any county in California.

We calculated cases per person-years for prisons X and Y based on data from a cohort of inmates who had spent >1 night in 2011 at either prison X or Y (study cohort). This cohort was subject to certain exclusions: inmates who spent time at both institutions during 2011, inmates who received a coccidioidomycosis diagnosis before 2011, and inmates who received a coccidioidomycosis diagnosis in 2011 at a prison other than X or Y. To derive the total number of person-years spent at the prisons, we summed the number of days each inmate was incarcerated at prison X or Y and divided that sum by 365.

### Risks for Primary Coccidioidomycosis

We defined primary coccidioidomycosis as an illness compatible with coccidioidomycosis that caused an inmate to seek medical attention and that was confirmed as coccidioidomycosis by a laboratory test. We collected primary cases from the CCHCS coccidioidomycosis surveillance system. We performed a cohort study to determine risk factors for primary coccidioidomycosis based on race/ethnicity, age, and whether the inmate had diabetes mellitus (DM); the latter was included in the model because studies and case series have identified an association between DM and complications of coccidioidomycosis (*6*–*9*). For this analysis, we again used the study cohort dataset that we had used to determine the coccidioidomycosis cases per person-years. Race/ethnicity, birthdate, and DM status were available for each inmate in the study cohort. Race/ethnicity is recorded on an inmate’s arrival into the CDCR prison system and is chosen by the inmate from a list of 27 race/ethnicities that includes a category called “other.” 

We grouped the race/ethnicity values into 5 categories: African American, Asian/Pacific Islander, Hispanic, white, and other. In the “other” category, we included all inmates who had self-identified as “other” on entry into prison, and all inmates who had self-identified as “American Indian.” We calculated age at midyear 2011 and included age as a continuous variable in our model. DM status of each inmate was determined on the basis of laboratory (hemoglobin A1C results) and pharmacy information (diabetic medication prescriptions). To determine risks for primary coccidioidomycosis, we explored interaction by using stratified analyses and then performed logistic regression on a model that contained all variables and interaction terms.

### Risks for Severe and Disseminated Coccidioidomycosis

To determine risk factors for severe and disseminated disease, we performed a case-control study. We defined severe disease as a case of coccidioidomycosis that was confined to the lungs (nondisseminated), for which the patient required >10 days of hospitalization. A patient was determined to have severe coccidioidomycosis if he was in the hospital for >10 days during which all hospital discharge International Classification of Disease, Ninth Revision (ICD-9) codes indicated nondisseminated coccidioidomycosis (ICD-9 codes 114.0 or 114.4–114.9). We defined disseminated coccidioidomycosis as disease in which the patient had a discharge ICD 9 code for disseminated coccidioidomycosis (ICD-9 codes of 114.1–114.3) for any hospitalization. Cases were derived from the CCHCS hospitalization discharge dataset for the period July 1, 2010, through April 11, 2013. This dataset contained hospitalization data for CDCR inmates incarcerated in any of California’s 33 adult prisons. Controls were patients in whom coccidioidomycosis was diagnosed in 2011 at prison X or Y who had not been hospitalized as of April 11, 2013. We evaluated the variables of race/ethnicity, age, and DM status by using logistic regression to predict severe and disseminated coccidioidomycosis. For the models for severe and disseminated coccidioidomycosis, our numbers were not robust enough to support models with interaction terms.

### Statistical Analyses

We used SAS 9.2 (SAS Institute; Cary, NC, USA) for all statistical analyses. The p value for statistical significance was set at ≤0.05.

## Results

### Coccidioidomycosis Incidence and Cases per Person-Years

For 2011, the coccidioidomycosis cases per 100,000 population for prisons X and Y were 6,934 and 3,799, respectively, 1–2 orders of magnitude higher than the rates at the other 6 prisons in 3 counties to which coccidioidomycosis was endemic (Table 1). These rates were also an order of magnitude higher than the 2011 rate for Kern County, and that difference was consistent over the period 2007–2012 (Figure).

**Table 1:** Coccidioidomycosis cases in prison X, prison Y, and prisons 1–6; in the communities surrounding the prisons X and Y and in Kern County, California; and in the state of California, USA, 2011

Location	No. cases	Mid-year population	Cases/100,000 population
Prison X	317	4,572	6,934
Prison Y	218	5,738	3,799
Prison 1	3	5,647	53
Prison 2	11	6,389	172
Prison 3	10	5,051	198
Prison 4	10	4,682	214
Prison 5	11	4,938	223
Prison 6	14	5,908	237
Communities			
City of prison X*	172	12,821	1,342
City of prison Y†	53	9,210	575
County of prison X	376	934,875	40
County of prison Y†	131	145,961	90
Kern County	2,568	848,958	302
California*†	4,607	37,559,818	12

*Prison X case and population counts were subtracted from these surrounding communities’ case and population counts. †Prison Y case and population counts were subtracted from these surrounding communities’ case and population counts.

**Figure:**
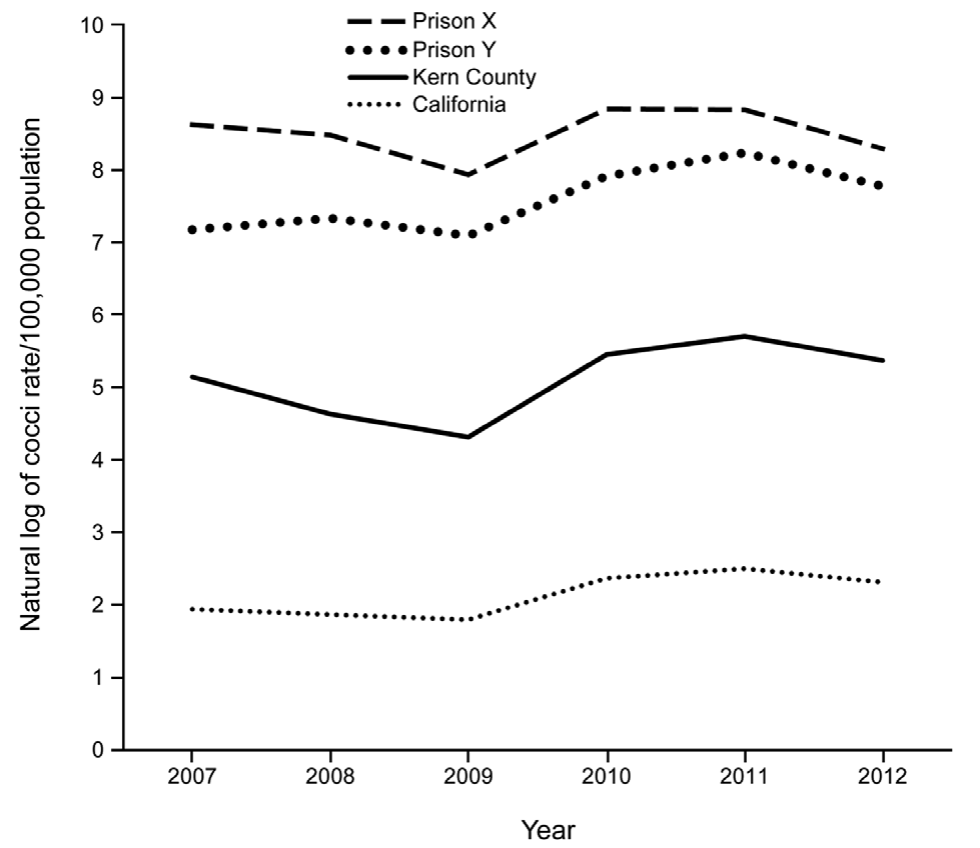
Natural log of coccidioidomycosis cases per 100,000 population for prison X, prison Y, Kern County, and the state of California, 2007–2012.

In 2011, 16,560 inmates spent at >1 night in prison X or prison Y. Of these, 834 were excluded from the analysis because they had been given a diagnosis of coccidioidomycosis before 2011 or at another institution in 2011; 155 were excluded because they were incarcerated at both institutions during 2011. Of the remaining 15,571 inmates, 6,064 inmates had been incarcerated at prison X and 9,507 at prison Y. In all, 516 had a diagnosis of coccidioidomycosis in 2011, 304 from prison X and 212 from prison Y. The 6,064 inmates of prison X spent 4,037 person-years at the prison, and the 9,507 inmates of prison Y spent 5,464 person-years at the prison. The coccidioidomycosis cases per 100,000 person-years was 7,530 for prison X and 3,880 for prison Y.

### Risks for Primary Coccidioidomycosis

Of the 15,571 inmates in the cohort, 6,558 (42%) were Hispanic, 4,380 (28%) were white, and 3,728 (24%) were African American. Asian/Pacific Islanders numbered 128 (1%), of which 36 were identified as Filipino. The remaining 777 (5%) inmates were categorized as other; of these, 183 (1% of the total cohort) were identified as American Indian. The median age of the inmates in the study cohort was 39 years (range 17–89 years; 10th percentile 24 years, 90th percentile 56 years).

In a univariable model, age was significantly associated with primary coccidioidomycosis (OR 1.009, 95% CI 1.002–1.016). Stratified analyses suggested an interaction between age and race/ethnicity in predicting primary coccidioidomycosis. We therefore created a model with variables for prison (prison X or Y), DM status, and the interaction term for age and race/ethnicity. Logistic regression on this model resulted in a significant association with primary coccidioidomycosis for incarceration at prison X (compared with incarceration at prison Y) and with days of incarceration at prison X or Y (Table 2). Also significant were African American and other race/ethnicity at >40 years of age and Hispanic race/ethnicity at >55 years of age. At age 55, African American, Hispanic, and other race/ethnicity were significantly associated with coccidioidomycosis with odds ratios of 2.5 (95% CI 1.7–3.6), 1.6 (95% CI 1.1–2.3), and 2.2 (95% CI 1.2–3.9), respectively, when compared to white persons (Table 3).

**Table 2:** Association of primary coccidioidomycosis with prison of incarceration, diabetes status, and the number of days incarcerated among inmates, California, USA, 2011*

Characteristic	No. (%) ill	No. (%) not ill	aOR	95% CI
Prison of incarceration				
Prison X	304 (58.9)	5,760 (38.3)	1.95	1.63–2.34
Prison Y	212 (41.1)	9,295 (61.7)	Referent	
Persons with diabetes	44 (8.5)	1,187 (7.9)	0.87	0.62–1.21
No. days at prison X or Y in 2011	NA	NA	1.007	1.006–1.009

*aOR, adjusted odds ratio; NA, not applicable: number of days incarcerated cannot be expressed as a single value for those ill and not ill.

**Table 3:** Association of race/ethnicity at 3 age points with primary coccidioidomycosis among a cohort of inmates incarcerated at prison X or Y, California, 2011

Characteristic	aOR*	95% CI
Race/ethnicity, age 25 y		
White	Referent	
African American	1.02	0.65–1.62
Hispanic	0.86	0.57–1.29
Asian/Pacific Islander	0.73	0.21–2.53
Other	1.26	0.65–2.44
Race/ethnicity, age 40 y		
White	Referent	
African American	1.59	1.23–2.06
Hispanic	1.18	0.92–1.51
Asian/Pacific Islander	0.93	0.33–2.58
Other	1.68	1.13–2.49
Race/ethnicity, age 55 y		
White	Referent	
African American	2.48	1.73–3.55
Hispanic	1.62	1.13–2.34
Asian/Pacific Islander	1.18	0.36–3.89
Other	2.23	1.23–3.92

*aOR, adjusted odds ratio.

### Risks for Severe and Disseminated Coccidioidomycosis

A total of 115 inmates had severe coccidioidomycosis, and 115 inmates had disseminated coccidioidomycosis (the equal numbers of severe and disseminated cases was coincidental). There were 474 prison X or Y inmates in whom coccidioidomycosis was diagnosed in 2011 who had not been hospitalized as of April 11, 2013. Logistic regression on a model containing DM status, race/ethnicity, and age resulted in a significant association between severe coccidioidomycosis and DM (OR = 3.2, CI = 1.8–5.8) (Table 4). Logistic regression on a model containing DM status, race/ethnicity, and age resulted in a significant association between disseminated coccidioidomycosis and African American race/ethnicity (OR = 1.9, CI = 1.1–3.4) (Table 5).

**Table 4:** Multivariable model for the prediction of severe coccidioidomycosis in inmates in whom coccidioidomycosis was diagnosed, California, 2011–2013*

Characteristic	No. (%) cases, n = 115	No. (%) controls, n = 474	aOR	95% CI
Diabetes	25 (21.7)	37 (7.8)	3.2	1.8–5.8
Race/ethnicity				
White	26 (22.6)	97 (20.4)	(ref)	
African American	41 (35.7)	152 (32.1)	0.97	0.5–1.7
Hispanic	39 (33.9)	189 (39.9)	0.80	0.5–1.4
Asian/Pacific Islander	1 (0.9)	3 (0.6)	1.47	0.1–14.9
Other	8 (7.0)	33 (7.0)	0.88	0.4–2.2
Age	NA	NA	1.001	0.983–1.019

*Cases represent patients requiring >10 days of hospitalization for nondisseminated coccidioidomycosis during July 1, 2010–April 11, 2013. Controls represent patients from prisons X and Y who received a diagnosis of coccidioidomycosis in 2011 but who had not been hospitalized as of April 11, 2013. aOR, adjusted odds ratio; NA, not applicable.

**Table 5:** Multivariable model for the prediction of disseminated coccidioidomycosis in inmates with coccidioidomycosis, California, USA, 2011–2013*

Characteristic	No. (%) cases, n = 115	No. (%) controls, n = 474	aOR	95% CI
Diabetes	9 (7.8)	37 (7.8)	0.82	0.4–1.8
Race/ethnicity				
White	19 (16.5)	97 (20.4)	Referent	NA
African American	57 (49.6)	152 (32.1)	1.92	1.1–3.4
Hispanic	32 (27.8)	189 (39.9)	0.90	0.5–1.7
Asian/Pacific Islander	1 (0.9)	3 (0.6)	1.92	0.2–19.7
Other	6 (5.2)	33 (7.0)	0.94	0.3–2.5
Age	NA	NA	1.010	0.992–1.028

*Cases represent patients requiring >10 days of hospitalization for nondisseminated coccidioidomycosis during July 1, 2010–April 11, 2013. Controls represent patients from prisons X and Y who received a diagnosis of coccidioidomycosis in 2011 but who had not been hospitalized as of April 11, 2013. aOR, adjusted odds ratio; NA, not applicable.

## Discussion

For >5 years, 2 California prisons for adult men experienced rates of coccidioidomycosis that exceeded the rate of Kern County by 1–2 orders of magnitude. Calculations of cases per person-years for these prisons for 2011 exceeded the cases-per-midyear population figures, further confirming the high rates of coccidioidomycosis in prisons X and Y. Various theories have been proposed to explain these high rates. During its investigation, CDPH explored the possibility that a change in provider practices in 2005 (e.g., increased testing for coccidioidomycosis) might have resulted in more diagnoses of coccidioidomycosis at prison X but found that no such change had occurred (J. Yuan, unpub. data). In 2013, we explored the possible contribution of a high population turnover (and thus frequent replenishment of susceptible persons) at prisons X and Y to the high rates, but we found no association (J. Mohle-Boetani, unpub. data). Most inmates at prisons X and prison Y resided in areas to which coccidioidomycosis was not endemic before incarceration, so their susceptibility to the disease is at least a partial explanation for high coccidioidomycosis rates compared to those of the surrounding communities. However, a naïve population does not explain the high odds for acquiring coccidioidomycosis at prison X (independent of age and race) compared to the odds at nearby prison Y. Nor does a naïve population explain the very high rates of coccidioidomycosis in the cities of prisons X and Y compared with their surrounding counties. Because coccidioidomycosis is not uniformly distributed even in the area to which coccidioidomycosis is endemic, the higher rates likely reflect either a higher concentration of ambient spores or a strain that is more pathogenic than strains found elsewhere.

Findings regarding the demographic and clinical risk factors from these analyses include the following: higher rates of primary coccidioidomycosis among persons >40 years of age are associated with certain races/ethnicity other than white; DM is associated with severe pulmonary coccidioidomycosis; and African American ethnicity is associated with disseminated coccidioidomycosis. These findings are not new, but have applications beyond the protection of this population. The association of African American ethnicity and disseminated coccidioidomycosis has been reported as early as 1945 (*6*,*10*,*11*) and is generally accepted among researchers and clinicians in the field. However, some authors refute the existence of a predilection for primary disease by race/ethnicity (*12*), even though an association between African American ethnicity and primary coccidioidomycosis has been reported by numerous investigators (*13*–*16*). We believe our finding of this association is substantiated because it is based on the study of a population with robust numbers of persons of non-white race/ethnicity. Moreover, inmates of all races/ethnicities are similar in their activities at the institutions, such as the time they spend in the yard. Equal and prompt access to health care for all inmates is a policy of the CCHCS administration (headed by a Federal Receiver) and is monitored by outside agencies. Our other findings with regard to increased risk for coccidioidomycosis and race/ethnicity, for example, that Hispanic inmates (>55 years of age) and those of other race/ethnicity (>40 years of age) are at higher risk than their white couterparts, are also consistent with the literature. Gifford calculated “coccidoidal granuloma” rates of Mexicans to be between those of whites and African Americans (*17*), as is the case for our population. Other race/ethnicity, in which we included those inmates who self-reported as American Indian, and those who self-reported as other, is not clearly defined, but may represent largely mixed-race persons. That inmates were in large enough numbers in the “other” race/ethnicity category to show a significantly increased risk for primary coccidioidomycosis compared with whites suggests the need to reevaluate the risk for mixed-race individuals. Other nonwhite races, specifically Asian-Pacific Islanders, should also be investigated further, because our numbers were insufficient to assess statistically significant associations.

A limitation of our study of primary coccidioidomycosis was that many of the non-ill inmates may have been previously infected and, therefore, immune to disease. Because no test for previous infection was available at the time of this study, we could not determine which inmates might have had asymptomatic infection in the past. However, this limitation would bias the findings toward the null, so does not negate our study findings. Another limitation is that inmates infected with coccidioidomycosis in their county of residence or in another prison may have had a diagnosis only after entering prison X or Y and were thus misclassified as exposed at these prisons. Although this could introduce a bias, we do not believe the acquisition of coccidioidomycosis outside of prisons X and Y would have considerable effects on our results. The numbers of cases are large for 2011, and our experience is consistent over many years that prisons X and Y report the highest coccidioidomycosis counts in our system.

On June 24, 2013, after review of the results of our analyses, and in consultation with court monitors and coccidioidomycosis experts, the United States District Court for the Northern District of California issued an order to exclude all African American inmates and inmates with DM from prisons X and Y (*18*). The order was enacted by CDCR.
